# The main findings in emergency computed tomography examinations

**DOI:** 10.1590/0100-3984.2024.57.e14-en

**Published:** 2025-01-10

**Authors:** Alexandre Bezerra

**Affiliations:** 1 Associate Professor of Radiology, Universidade de Brasília (UnB), Brasília, DF, Brazil. Email: alexbezerra@gmail.com.

The use of diagnostic imaging methods, especially computed tomography (CT), has increased significantly in recent years^(1,2)^. In Brazil and in other countries, that increase has occurred in the context of outpatient care and emergency care^(3,4)^. In hospital emergency departments, there has been an increase in the use of CT in trauma victims and in patients with other conditions that warrant emergency care, such as abdominal pain and dyspnea^(5,6)^. In addition, the recent pandemic of coronavirus disease 2019 has led to an increase in the use of chest CT in emergency departments, because there was a need to quantify the pulmonary involvement of these patients. This increase in the number of CT scans, which generates an increase in health care costs and increases patient exposure to ionizing radiation, has raised questions about its possible causes, such as a lack of confidence on the part of less experienced doctors, a desire to shield against medical malpractice lawsuits, and even patient expectation and desire to undergo imaging examinations^(7-9)^.

There are few data in the literature on the most common findings on CT examinations ordered in hospital emergency departments^(5,6)^. This lack of data is much more evident in the case of examinations unrelated to trauma. The study conducted by Soares et al.^(10)^, recently published in Radiologia Brasileira, seeks to bridge that gap and investigates precisely the main findings on CT of the head, chest, and abdomen in examinations of adult patients, admitted for traumatic or non-traumatic emergencies, at a tertiary care hospital in Brazil. The most frequently performed examination was CT of the head, followed by CT of the abdomen and CT of the chest, in keeping with data in the literature^(2,5)^. Notably, the authors demonstrated that pathological findings were observed in less than a third of the CT scans of the head performed in non-traumatic emergencies, the most common being those consistent with stroke. They also found that pathological findings were observed in approximately two thirds of the chest CT scans performed in non-traumatic emergencies, with pneumonia being the most common such finding, whereas pathological findings were observed in less than one third of those performed in traumatic emergencies, among which pulmonary contusion and bone lesions were the most common findings. On abdominal CT scans performed outside the context of trauma, urinary lithiasis was the most common finding. One interesting finding of the study is that pathological findings were observed in only approximately 11% of the abdominal CT scans performed within the context of trauma. It is possible that performing full-body CT scans in traumatic emergencies might explain, in part, the data presented by the authors. Although there are few data on pathological findings from CT scans performed in the emergency department, there have been various studies of incidental findings from CT scans performed in that setting^(11,12)^.

One limitation of the Soares et al.^(10)^ study is that the authors did not present the clinical data that motivated the examinations (e.g., abdominal pain, neurological signs, or cough) or in which clinical conditions the rate of pathological findings was highest. These data could be the subject of future investigations for comparison with data from other countries^(5,6)^. The authors also did not report how many full-body CT examinations were performed in cases of trauma within the study sample.

The data presented in the Soares et al.^(10)^ study are extremely important for the development of institutional protocols aimed at rationalizing and guiding the examinations ordered by attending physicians in emergency departments. Various criteria for ordering imaging examinations have been proposed, such as the American College of Radiology Appropriateness Criteria and the Brazilian Medical Association Guidelines Project, as well as the UK National Institute for Health and Care Excellence guidelines, the New Orleans Criteria, and the Canadian CT Head Rules, in the context of trauma. Knowledge of data specific to Brazil could help emergency physicians in the country make better decisions when faced with the diverse cases seen in emergency departments.
